# Privacy-aware sharing and collaborative analysis of personal wellness data: Process model, domain ontology, software system and user trial

**DOI:** 10.1371/journal.pone.0265997

**Published:** 2022-04-07

**Authors:** Lauri Tuovinen, Alan F. Smeaton

**Affiliations:** 1 Biomimetics and Intelligent Systems Group, University of Oulu, Oulu, Finland; 2 Insight SFI Research Centre for Data Analytics, Dublin City University, Dublin, Ireland; King Abdulaziz University, SAUDI ARABIA

## Abstract

Personal wellness data collected using wearable devices is a valuable resource, potentially containing knowledge that goes beyond what the device and its the associated software application can tell the user. However, extracting such knowledge from the data requires expertise that an average user cannot be expected to have. To overcome this problem, the data owner could collaborate with a data analysis expert; for such a collaboration to succeed, the collaborators need to be able to find one another, communicate with one another and share datasets and analysis results with one another. In this paper we presents a process model for such collaborations, a domain ontology and software system developed to support the process, and the results of a user trial demonstrating collaborative analysis of sleep data. Unlike existing collaborative data analytics tools, the process and software have been specifically designed with the non-expert data owner in mind, enabling them to control their data and protect their privacy by selecting the data to be shared on a case-by-case basis. Theoretical analysis and empirical results suggest that the process and its implementation are valid as a proof of concept.

## Introduction

Personal data is gathered by individuals or by third parties, in the first instance to improve some aspect of the individual’s life. This may be to do with their health and wellness [1], for example sleep or physical activity levels or nutritional intake, or to do with their online activities such as measuring their screen time on mobile devices [2]. Personal data is also widely regarded as a valuable business asset that companies can monetize e.g. by creating new or improved services, by improving the targeting of advertisements, or by trading the collected data [3]. In the case of social computing platforms, the monetary value of data has become the foundation of the business models of many of them, which are typically available to regular users free of charge; from the individual user’s perspective, their data becomes a kind of implicit currency that can be used to gain access to services offering desired features such as the ability to communicate with other people and share content with them [4].

Public discourse on the collection and exploitation of personal data largely revolves around the model where the collection and exploitation is done by organizations while the role of the individuals is to passively allow this to happen. When the individuals are considered as active agents, typically the focus is on how they can protect their data against collection by organizations they do not trust or against exploitation for purposes they do not approve. As a concrete manifestation of this way of thinking, we may look at data protection legislation such as the General Data Protection Regulation (GDPR) of the European Union [5], which distinguishes between data *subjects*, i.e. natural persons who are granted various rights regarding their personal data, and data *controllers* and *processors*, i.e. organizations that are required to fulfill various obligations in order to be legally permitted to process such data.

This type of discourse, while certainly important, overlooks the interesting possibility of individuals themselves collecting their own data and exploiting it themselves for their own personal benefit. One type of data that many people are already collecting and using for their own purposes is physiological data representing a person’s physical activity and sleep. Data like this can be recorded using a variety of consumer-oriented wearable and ambient sensor devices, many of which are relatively inexpensive as well as easy to use and therefore accessible to a large number of potential users. From the device, data is typically transferred wirelessly to a personal computer or mobile device, where it can be viewed using an application supplied with the device. The application may also upload the data to a server where it can be accessed via an online dashboard using a standard web browser.

Data like this, assuming that it is at least reasonably accurate, is potentially of considerable value for the person collecting it, because it enables them to make better-informed decisions concerning matters that are highly relevant to their own physical and mental well-being. Its value could be further enhanced by analyzing it e.g. to reveal interesting temporal patterns such as trends and periodicities; additional insights may be obtained by combining it with data from another source or device. However, there are factors that make this difficult for the average person to achieve, one of which is that the data is typically controlled by the company that made the device with which the data was collected, and the controller may or may not enable the user to easily export their data in a portable and re-usable format.

In many cases, data collected using personal wellness products can be exported from a website with relatively little effort, but this in itself does not help unless the user also has the skills required to analyze the data. Most people do not have such skills, nor can they be realistically expected to acquire them. However, if the owner of the data is able to find someone who does have the required skills and is willing to help, the two can collaborate to refine the data into knowledge that the data owner is interested in. To support such collaborations, we have developed an experimental online software platform that enables data owners and data analysts to find one another, communicate and exchange datasets and analysis results over the Internet.

Our software platform consists of client and server applications and server- and client-side databases. Internally, the platform uses a domain ontology as a shared data structure through which the collaborations are mediated. To maximize the control of data owners over their datasets, the platform employs a decentralized data management architecture where the datasets are stored on the users’ own devices instead of the server host. When sharing data with a collaborator, the data owner can create privacy constraints to specify that the collaborator should have access only to specific parts of the dataset; the underlying ontology and the reasoning capabilities enabled by it allow such constraints to be represented and enforced.

To test the validity of the concept of collaborative analysis and the ability of the platform to support it, we conducted a trial in which a number of volunteers collected sleep data using wearable sensor devices and used the platform to study this data in collaboration with others who were data analysts in the trial. The trial demonstrated the fundamental viability of the ontology-based architecture. Furthermore, the results of a feedback survey completed by participants after the trial indicate that the concept and process of collaborative analysis of personal data using a platform wity the characteristics of the one used here are valid and should be studied further.

The principal contributions of the paper are as follows:

- A model for the process of collaboration between a non-expert data owner and an expert data analyst for extraction of knowledge from the data owner’s personal data;
- A description of the functionality, architecture and implementation of a software platform developed to facilitate such collaboration;
- A report and analysis of the results of a trial carried out as a proof-of-concept of the above.

In the remainder of the paper, we first present essential background information and review related work. We then discuss the concept, process, tasks and challenges of collaborative personal data analytics. This is followed by a description of the functionality, architecture and implementation of the collaboration platform. Finally, we present our validation results and discuss them critically before concluding the paper.

## Background and related work

Terms such as lifelogging [6] and the quantified self [7] have been used to refer to the practice of an individual using technology to collect, archive and analyze data representing various aspects of their own life. A wide range of personal sensor devices may be used to collect such data, and there is likewise a wide range of potential applications for such data. However, many of these have had a limited impact among the general populace; for example, using a wearable camera such as SenseCam [8] to automatically capture a continuous stream of images remains a niche activity.

One form of personal data collection that has become notably popular is activity and sleep tracking. According to a survey carried out in 2019, 21% of American adults regularly wear a fitness tracker or smart watch [9]. The products available for this purpose include a diverse range of wearable devices, ambient sensors (placed under the mattress or on a bedside table, for example) and mobile software applications (using sensors commonly included in smartphones, such as accelerometers and microphones). Some of these only provide an interface for viewing the data, making them unsuitable for collecting data to be analyzed using a different application, but there are many that allow a user to export the data in a portable format such as CSV, enabling the user to import the data into any of a large number of data analysis tools.

A basic wearable fitness tracker capable of recording data on the user’s activity and sleep can now be purchased for well under 100 USD, and some mobile applications can even be downloaded free of charge. Several powerful data analysis tools are also available free of charge, so the main obstacle to the collection and analysis of personal data by the average individual is not the financial investment but the required expertise. In theory, expertise can also be acquired, but in practice, there are several factors that may make this an unworkable option. If this is the case, the natural option remaining is to collaborate with someone who has the necessary data analytics expertise and is willing to assist.

Supporting collaborations like this requires tools that enable the collaborators to communicate, share and edit various artifacts over distance, both synchronously and asynchronously. General-purpose communication and collaboration tools are available and widely used, but a platform designed for the specific purpose of supporting collaborative analysis has many advantages. Several such platforms can be found in the literature, and there are also several available as commercial services on the Web.

Perhaps the single most studied aspect of collaborative data analysis is collaborative visualization and interpretation of data, with a large number of papers exploring the conceptual and architectural issues involved (e.g. [10–14]), as well as a similarly large number of proposals for how collaborative visualization can be done effectively using a variety of display equipment ranging from conventional two-dimensional screens to immersive environments such as CAVEs (e.g. [15–20]). Another relatively narrow perspective on collaborative analysis is to consider only the sharing of resources among the collaborators; in this approach, collaborators have access to a shared repository, from which they can retrieve resources contributed by others and to which they can contribute resources for others to use, but the application of resources is up to each collaborator individually. Typically the repository is used to share data, as in systems such as Google Fusion Tables [21], SQLShare [22], DataHub [23] and SeedMe [24]. It is also possible to collaborate by sharing data analytics tools and workflows, as is the case with RCloud [25], KDDVM [26, 27] and the collaborative big data analytics platform of [28].

Proceeding towards more generic systems for online collaborative data analysis, there are several that are not so limited in terms of the collaborative activities they support, but are designed to be used in a specific application domain. Resource sharing is still an important element of collaboration in such systems, but they go beyond being mere repositories by providing special environments for the collaborative refinement of ideas into data analyses. Often the application domain addressed is some field of natural science—see, for instance, BSC [29], Dicode [30] and Galaxy [31] for the life sciences, and CyberSKA [32] and VISPA [33] for the physical sciences—but there is also, for example, the SWAB tool [34] for collaborative analysis of social media data. Cytomine [35] specializes not in a particular application domain but a particular type of data, namely multi-gigapixel images.

Finally, a number of existing systems are both generic in terms of collaborative functionality yet agnostic in terms of the application domain in which they are used. In the most straightforward case, a system provides a collaborative environment for the development of data analytics applications using a specific tool or a small set of tools. The most obvious choices of tool are a scientific computing software package, as in the case of Biocep-R [36], or a programming language with a good supply of available data analytics libraries, as in the case of Mode [37]. However, there is also a proposal for the use of linked spreadsheets as a platform for collaborative data analytics [38].

Another category of generic collaboration systems uses data analytics notebooks as the principal instrument of collaboration. The notebook in these systems is analogous to a traditional laboratory notebook, but it can be edited from anywhere and may contain a variety of complex digital objects, including code snippets and analysis results. For the execution of the analysis code, interfaces to several different data analytics tools are typically provided, allowing the users to select those that suit their tastes and needs. Examples of systems that employ the notebook approach include LabBook [39], Apache Zeppelin [40], Dataiku [41] and Databricks [42].

A third category of general-purpose collaborative data analysis covers systems where application development is based on the construction of knowledge discovery workflows. These systems provide a graphical editor with a drag-and-drop style user interface. Using the interface, users can select components representing various data transformation operations and place connectors between them to specify the flow of data through the application. Systems that allow this to be done collaboratively include KD-ASP [43], KDDesigner [44] (from the authors of KDDVM and part of the same ecosystem), ShareInsights [45] and Dataiku, which features both notebooks and a workflow-based interface.

Yet one more group of online collaborative systems worth mentioning are those designed to support research activities, such as Synapse [46]. These incorporate features of collaborative data analysis systems for the purpose of managing and analyzing research data, but also provide tools to support other aspects of research work such as authoring scientific publications. In terms of our classification, these might be best categorized as notebook-based systems, but the fit is not perfect. Other ambiguities can be pointed out as well, as DataHub, for instance, provides a complete notebook-based data analytics environment and could thus be considered a generic system, but its notebooks are not themselves collaborative, only the dataset sharing and versioning functionality is.

Despite the wide variety of systems available, there do not seem to be any designed specifically to support collaboration on equal terms between experts and non-experts. There are various crowdsourcing systems where non-experts can be engaged to contribute to data analysis projects by providing access to computing resources (e.g. [47, 48]) or performing relatively simple manual tasks (e.g. [49, 50]), but this collaboration concept has little in common with the one we are discussing, where it is primarily the non-expert who provides the data and determines the analysis objectives. In the next section we examine this concept, and the special requirements its implementation entails, in more detail.

## Collaborative data analytics

### Concept and process

The concept of collaborative data analysis which is the focus of this paper differs considerably from the kind of collaboration for which existing systems mentioned in the previous section are intended. The key differences are twofold:

1. The collaboration is driven by the needs and interests of an individual who cannot be assumed to have any particular technical expertise, making it challenging to define a model of collaboration that incorporates them as equal partners.
2. The data analyzed is personal and potentially sensitive in nature, making it essential that the collaboration process include mechanisms for resolving privacy issues.

Given this premise, focusing on facilitating collaborative development of data analytics code or workflows is not the best option, because the owner of the data is likely to have limited ability (or none) to make any meaningful contributions to such work. Supporting this type of collaboration therefore requires that we revise the way of thinking on which the design of the existing systems is based.

A data owner with no knowledge of data analysis may still have ideas concerning what sort of knowledge they would like extracted from their data, but without expertise it is not possible to have a clear understanding of how to get from the data to the desired results, and it may even be difficult to formulate concrete objectives. The collaboration can therefore be described as a process whereby the data and ideas of the data owner are refined first into specific questions that can be answered by analyzing the data and then into answers to those questions. At the heart of the process is an ongoing dialogue between the collaborators, and it is this dialogue that the collaboration platform should facilitate.

**Fig 1:**
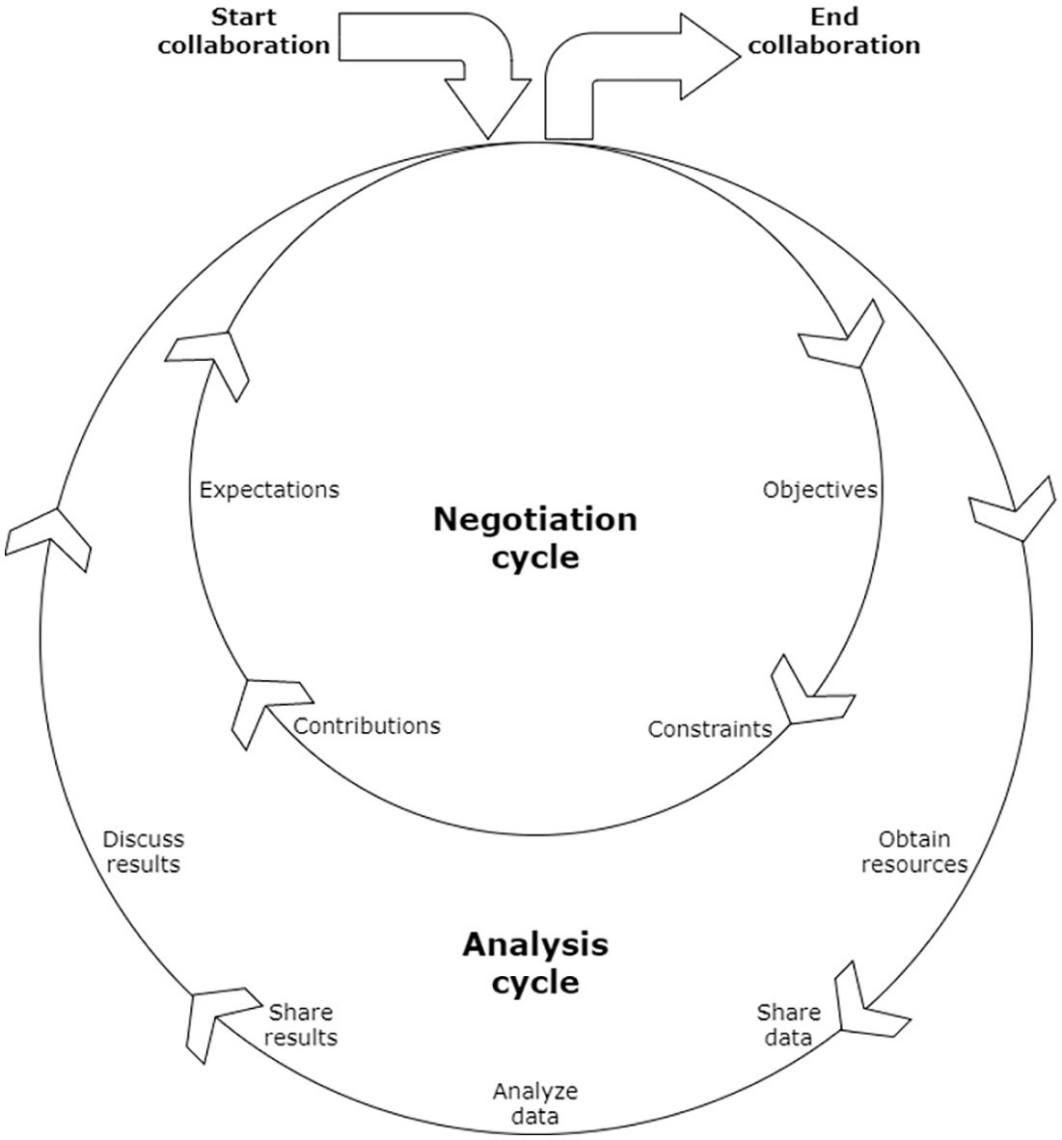
The collaboration process. The process of collaborative analysis of personal data can be modeled as a sequence of two cycles. In the negotiation cycle, the collaborators agree on the terms of the collaboration; in the analysis cycle, they pursue the objective defined in the analysis cycle.

More formally, we can describe the collaboration process as an alternation of two cycles, illustrated in Fig 1. These are the *negotiation* cycle and the *analysis* cycle, representing two different types of dialogue between the data owner and the expert. The cycles can be described as follows:

- In the negotiation cycle, the collaborators aim to come to an agreement on the terms of the collaboration. This involves discussing the objectives of the collaboration, the constraints to be observed while pursuing the objectives, and the contributions and expectations of each collaborator.
- In the analysis cycle, the collaborators aim to achieve the objectives defined in the negotiation cycle. This involves determining and obtaining access to required resources (e.g. data analysis tools or supplementary data sources), exchanging input data and analysis results, and discussing the significance of the results in terms of the objectives.

**Fig 2:**
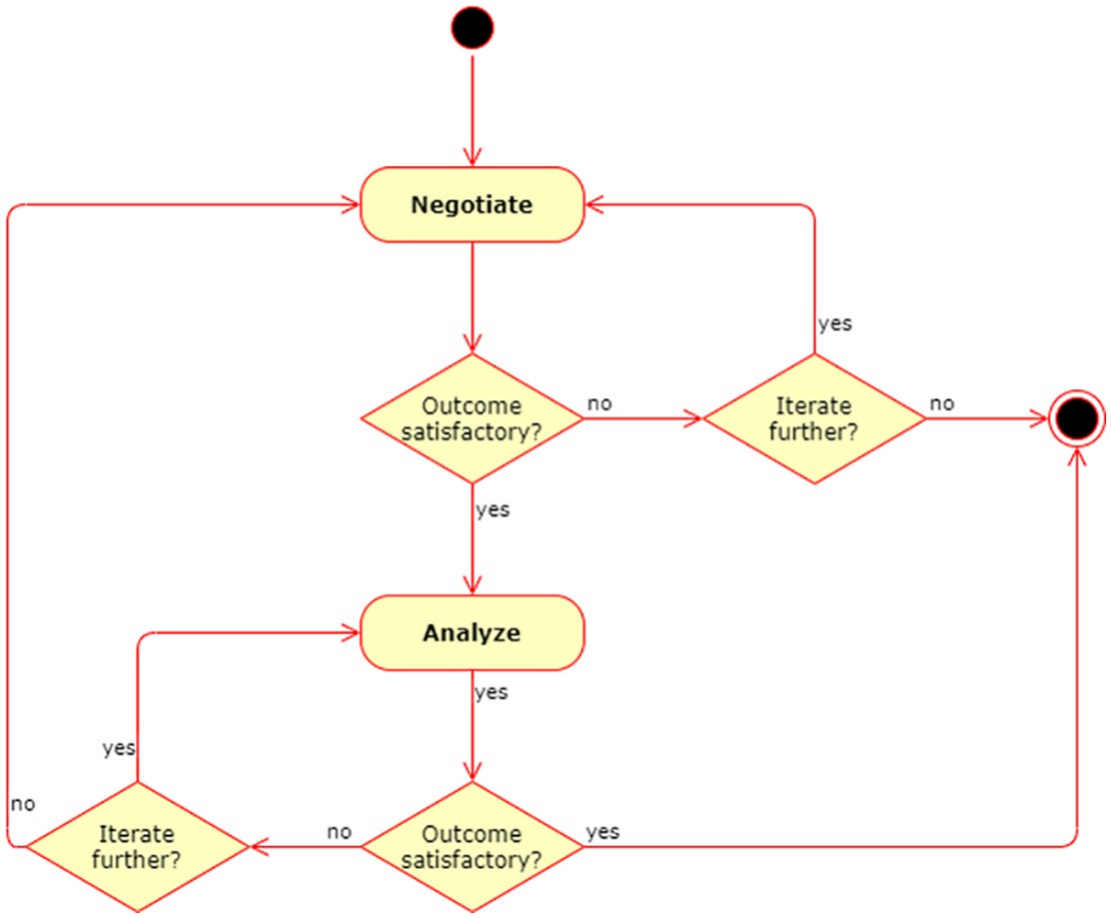
Transitions between cycles. A decision is taken at the end of each execution of a collaboration cycle. This can lead to a repeat of the same cycle, a transition to the other cycle or the conclusion of the collaboration.

Each execution of each of the cycles is followed by a situation evaluation where the next course of action is determined. In either case the options are to repeat the cycle, to proceed to the other cycle or to end the collaboration, so any sequence of the two cycles is possible. This is illustrated in Fig 2.

### Tasks and challenges

Before the collaboration can begin, the collaborators need to be brought into contact with one another. Assuming that the collaboration is initiated by the data owner, they need to be able to search for potential collaborators with expertise in areas relevant to the data owner’s interests and the data they have collected. Having identified a candidate (or candidates) whose expertise matches the requirements, the data owner can invite them to join the collaboration. To enable this, the collaboration platform must maintain a profile of each expert and make the profile information discoverable using tools that are accessible to non-expert users.

When the collaboration has been initiated, the participants can begin the first negotiation cycle. To facilitate negotiation, the collaboration platform must at least provide a communication channel that the collaborators can use to talk to one another. However, this is just the minimum level of support; ideally, the platform should assist the collaborators in achieving a mutually acceptable negotiation result. This can be done by implementing functionality for representing and analyzing the collaborators’ contributions and expectations, enabling the platform to detect incompatibilities and guide the collaborators toward potential resolutions.

A concrete example of where support for conflict resolution may be needed is preserving the data owner’s privacy, or rather finding an acceptable trade-off between data sharing and privacy preservation. The data requirements of the analysis to be carried out provide the baseline, but the data owner may wish to impose constraints depending on their privacy preferences and the level of trust that exists between the collaborators. On the other hand, the expert may have an interest in the data that goes beyond what is required to satisfy the interests of the data owner; gaining access to the data may well be part of why the expert is willing to participate in the collaboration.

With multiple factors affecting the negotiation and pulling in different directions, it is not necessarily easy to reach an agreement that preserves both the utility of the data and the privacy of the data owner to a degree that is acceptable to everyone involved. The collaboration platform should facilitate the process of finding such an agreement by identifying conflicting expectations and suggesting trade-offs that may be adopted to resolve such conflicts. Particularly the data owner is likely to require support, because some expertise is required to fully understand the utility and privacy implications of the decision to share or withhold a given set of data.

Assuming that a mutually satisfactory negotiation result is eventually achieved, the data owner will now need to share the data with the expert to begin the analysis cycle. To support this, the collaboration platform needs to provide data management functionality, enabling data owners to integrate data sources and organize their data into collections. Data selected to be shared with collaborators needs to be made accessible to those collaborators while protecting its confidentiality against access by any unauthorized third parties. The data may also need to be transformed in various ways; for instance, it may be desirable to reduce the sensitivity of a dataset by performing a privacy-enhancing transformation before the data is shared.

To support the analysis of the shared data by an expert (or experts), the collaboration platform must enable the data to be processed using data analysis tools. This can be done by implementing a suite of tools as part of the platform, by enabling the use of external tools, or both. From a privacy standpoint, it would arguably be preferable to prevent the exportation of shared data out of the platform, which would make implementing analysis tools as part of the platform the only possible solution. On the other hand, the expert would probably prefer to use whichever tools they consider the most effective for the job at hand. If both internal and external tools are available, the data owner can be given the choice of allowing or disallowing exportation when sharing their data.

Here we should consider the possibility of multiple experts participating in the same collaboration and working on the same data. In this case it would be beneficial if the analysis tools used, whether internal or external, are collaborative, providing functionality similar to that of the collaborative analytics platforms surveyed in the previous section. Ideally, the tools should also enable the (non-expert) data owner to contribute, but how this might be facilitated effectively is a research question that is outside the scope of this paper.

Finally, when the expert has analyzed a batch of data and produced results that are potentially of interest to the data owner, the results need to be communicated to the data owner in a format that can be understood by a person with no technical expertise. To support this, the collaboration platform should enable the expert to create visualizations of the results and present them to the data owner. Preferably the visualization capabilities should also be collaborative, with features such as synchronous collaborative exploration and annotation of the analysis results.

## The collaboration platform

### Key requirements

The central functional requirements of the collaboration platform were derived from the tasks and challenges discussed in the previous section. We thus specified a system with the following key features:

- finding potential collaborators based on their expertise;
- communicating with collaborators;
- creating and managing datasets;
- sharing data with collaborators;
- creating and visualizing analysis results.

The most important part of the functionality of the platform is the management and sharing of datasets. There are a few special requirements associated with this, arising from the nature of this type of collaboration. The process of collaborative personal analytics driven by the data owner: the data processed and the analysis tasks carried out in a given collaboration are ultimately determined by what data the owner has collected, what the owner is willing to share with others and what kind of knowledge the owner is interested in. The data owner should therefore have control over where, when and how the data is stored and processed, insofar as this is practical.

From this guiding principle, three major data management requirements for the collaboration platform were derived. The first one of these is that there should be **no centralized storage of user data**. Instead of users storing their datasets on the collaboration server, they should be able to retain the data locally and only upload it when sharing it with another user. Besides reducing the vulnerability of the data, this also considerably reduces the storage capacity requirements of maintaining an instance of the platform.

The second requirement is that when sharing data with another user, a user should have the ability to **specify the details on a case-by-case basis**. For any given dataset, it should be possible for the owner to specify the part of it to be shared individually for each collaboration and collaborator, and furthermore, they should have the option of first transforming the data to reduce its sensitivity. Regardless of the data owner’s level of technical expertise, it should be convenient to use these facilities and easy to understand what part of the dataset is going to be shared in each case.

The third requirement concerns datasets produced by expert collaborators by processing data shared with them by a data owner. According to the principle of the data owner being in control, the owner of the original input dataset should also have access to and control over any datasets derived from it. To enable this, the collaboration platform must **maintain dataset provenance** by tracking the creation of derivative datasets. The following subsections describe how these requirements are addressed in the design of the platform.

### Functionality

From the user’s perspective, our collaboration platform has two interface levels: the main level and the collaboration level. The main level is what is first presented to the user after logging in, and its functionality is divided into four sections, represented as tabs in the main window. These are collaborations, data management, profile and invitations.

In the collaborations section, the user can create new collaborations as well as manage their existing collaborations. Information about ongoing collaborations can be viewed here, including the participants and datasets involved in each collaboration. Participants can be added and removed by the leader of a collaboration (i.e. the user who created it), whereas datasets can be added and removed by their owners in any collaboration that they are participating in.

In the data management section, the user can see all the datasets they have access to, including both datasets owned (created) by the user and those that have become accessible to the user through collaborations. New datasets can be created here, and data can be imported into existing datasets owned by the user. Data can also be exported into files from any datasets that the user has access to.

In the profile section, the user can view and edit their personal details. These include their login information (username and password), real name (or the name they want other users to see), profile text and expertise. Areas of expertise are stored in the underlying ontology and described with keywords; when specifying their areas expertise, the user can choose from those already available in the ontology or create new ones. Any number of areas of expertise may be specified for a given user.

In the invitations section, the user can view and manage their collaboration invitations, both ones sent to other users and ones received from other users. Received invitations can be responded to (accepted or rejected) here. To send an invitation, the user first performs a keyword search for users with expertise in the desired areas; the platform then returns a ranked list of candidates for the user to choose from. An optional message can be attached to the invitation before it is sent to the expert.

The collaboration level functionality of the platform can be accessed by selecting a collaboration in the collaborations section of the main window and opening it. This launches a new window where the user can perform operations pertaining to the selected collaboration. The functionality available here can be divided into three categories: collaboration data management, visualizations and chat.

The main sub-functions of collaboration data management are processing of data requests and creation of derivative datasets. Data requests are the way by which users of the platform can obtain access to datasets owned by their collaborators. The owner of the requested dataset may grant or deny the request; optionally, privacy constraints can be attached to a granted request to limit the access of the requesting user to specific parts of the dataset. Derivative datasets are a special category of dataset created by processing another dataset; a dataset designated as a derivative automatically becomes accessible to the owner of the original input dataset, so there is no need to create a data request in this case.

Visualizations are used to provide graphical representations of analysis results. Visualizations are created by selecting a dataset to visualize and launching a visualization editor, where the parameters of the visualization can be set and the appearance of the visualization previewed. Once a visualization has been created, all collaboration participants who have access to the underlying dataset may view it.

A chat function is available to all collaboration participants, enabling them to discuss any issues relevant to the collaboration. When launched, the chat opens in a separate window. The entire chat history since the beginning of the collaboration is available to all collaborators, and the names of collaborators currently logged in to the platform can be seen in a corner of the chat window.

Using the functionality provided by the platform, both cycles of the collaboration process outlined in the previous section can be executed. With the expert search and invitation functions, a data owner can find a suitable partner to collaborate with, and with the chat the collaborators can discuss the specifics. The result of the negotiation can be enacted through the creation of data requests and privacy constraints, after which the expert will be able to download the shared data and export it from the platform for analysis.

Since the data analysis takes place outside the platform, the expert is free to use whatever tools they deem best for the task. Having analyzed the data, the expert can import the results as derivative datasets, enabled by the same data import function that the data owner used to create the original dataset. The expert can then create visualizations to illustrate the results and use the chat to explain the visualizations. Further details, including screenshots of the platform in action, can be found in [51].

### Domain ontology

The domain ontology underlying the platform serves two purposes: to provide a shared data structure for representing objects involved in collaborations, and to serve as a repository for domain knowledge that the platform can use to assist the users in their tasks. The ontology fills a gap at the intersection of related domains, particularly data mining and privacy, for which domain ontologies (and semantic technologies in general) have previously been developed (e.g. [52–58] for data mining / knowledge discovery, [59–65] for privacy). The most important classes of the ontology, as used by the platform, are listed in Table 1. Further details on the ontology can be found in [66]; below we explain how the ontology addresses the data management requirements, which has not been discussed in previous publications.

**Table 1:** Main classes of the collaboration ontology.

Class	Explanation
Collaboration	Represents a collaborative data analysis project
Collaborator	Represents a user of the collaboration platform
Dataset	Represents a collection of data, possibly containing other datasets as subsets
DataItem	Represents an single piece of data, such as an individual variable value
Expertise	Represents an area of expertise that can be used to find a potential collaborator
Invitation	Represents an invitation to collaborate sent to a potential collaborator
DataRequest	Represents a request to share a Dataset sent to a collaborator
PrivacyConstraint	Represents an access restriction created by the owner of a Dataset in response to a DataRequest
Visualization	Represents a graphical representation of a Dataset, typically one containing analysis results

Most types of information processed by the platform are represented internally as individuals in the ontology and their properties. Collaborative analysis projects are represented in the ontology as individuals of class **Collaboration**, and the participants of such collaborations as individuals of class **Collaborator**. Collections of data are represented as individuals of class **Dataset**; Datasets may contain other Datasets as subsets, representing, for example, individual variables within the Dataset. Datasets not consisting of other Datasets consist of **DataItems**, representing individual pieces of data, typically values of a variable.

A data analysis expert, represented by the Collaborator subclass **Expert**, can request a Dataset to be shared by creating a **DataRequest**. When responding to the request, the owner of the Dataset, represented by the Collaborator subclass **DataOwner**, can create **PrivacyConstraints** to deny the Expert access to specific parts of the Dataset. The scope of a PrivacyConstraint can be specified in terms of individual DataItems, but more typically in terms of **DataGroupings**. The DataGrouping class has two subclasses, Dataset and **DataCategory**, the latter representing a grouping of DataItems based on some shared attribute. Similar to Datasets, DataCategories may contain other DataCategories as subcategories. DataItems asserted as members of a given DataGrouping are inferred to be members of all of its known supergroupings; via this inference the individual DataItems in the scope of a PrivacyConstraint can be identified.

An extension of the ontology introduces the **AnalysisTask** class, representing an individual operation specified by an Expert to be carried out as part of a broader effort to extract knowledge from a Dataset, such as extraction of features or application of an analytics model. An AnalysisTask may consist of other AnalysisTasks as subtasks; each AnalysisTask not broken down into subtasks requires access to some DataItems and uses some **AnalysisMethods**, representing the computational algorithms to be applied to the data in order to complete the task. As the result of the completion of an AnalysisTask, new Datasets are generated; these are inferred to be derivatives of the Datasets from which the input DataItems came from, enabling the provenance of a given Dataset to be traced back along the chain of derivation.

In the extended ontology, AnalysisTasks function as implicit DataRequests that may be limited by PrivacyConstraints. Each PrivacyConstraint prescribes an **AccessRestriction**, representing an operation to be carried out on the requested data before it is released. An extreme case of this is where access is denied altogether, but more permissive restrictions are possible by having the data undergo a transformation that reduces its sensitivity while retaining some of its utility. Algorithms used to perform such transformations are represented by the **ProtectionMethod** class, the privacy-preservation counterpart of the AnalysisMethod class. A ProtectionMethod could be, for example, a specific way of aggregating DataItems to generate a more abstract (and therefore less sensitive) representation of the Dataset containing them.

Like AnalysisTask and AnalysisMethod, AccessRestriction and ProtectionMethod are new classes introduced by the extended ontology. One more new class is **UtilityReduction**, representing the negative effect of a given ProtectionMethod on the performance of a given AnalysisMethod. This may be different for each individual kind of AnalysisMethod, enabling the characterization of ProtectionMethods and AnalysisMethods in terms of how compatible they are with one another. Furthermore, each ProtectionMethod and AnalysisMethod can be designated as being a substitute for other ProtectionMethods and AnalysisMethods, respectively. This enables the enumeration of all valid combinations of ProtectionMethod and AnalysisMethod and the selection of the combination that results in the least UtilityReduction, provided that the UtilityReductions can be meaningfully quantified.

### Architecture and implementation

The collaboration platform consists of client and server components, both of which are implemented as desktop software applications. Each individual user of the platform runs an instance of the client component, which communicates with the single-instance server component over a TCP/IP connection with TSL encryption. All communications among clients are mediated by the server.

The server component consists of a software application, a database and a master ontology. The application handles client sessions and has a graphical user interface that displays status information and allows the server administrator to configure the server and manage user accounts. The database stores the users’ login credentials and chat messages; is is also used to maintain a log of server events and to temporarily store datasets being transferred from one user to another.

Apart from credentials, all information about the users of the platform is stored in the master ontology as properties of the corresponding Collaborator individuals. Similarly, information about collaborations and datasets is stored in the ontology as individuals and properties, and the client and server applications enable the users to collaborate by providing an interface by which they can synchronously manipulate the contents of the ontology. The server component is responsible for maintaining the ontology in a consistent state.

**Fig 3:**
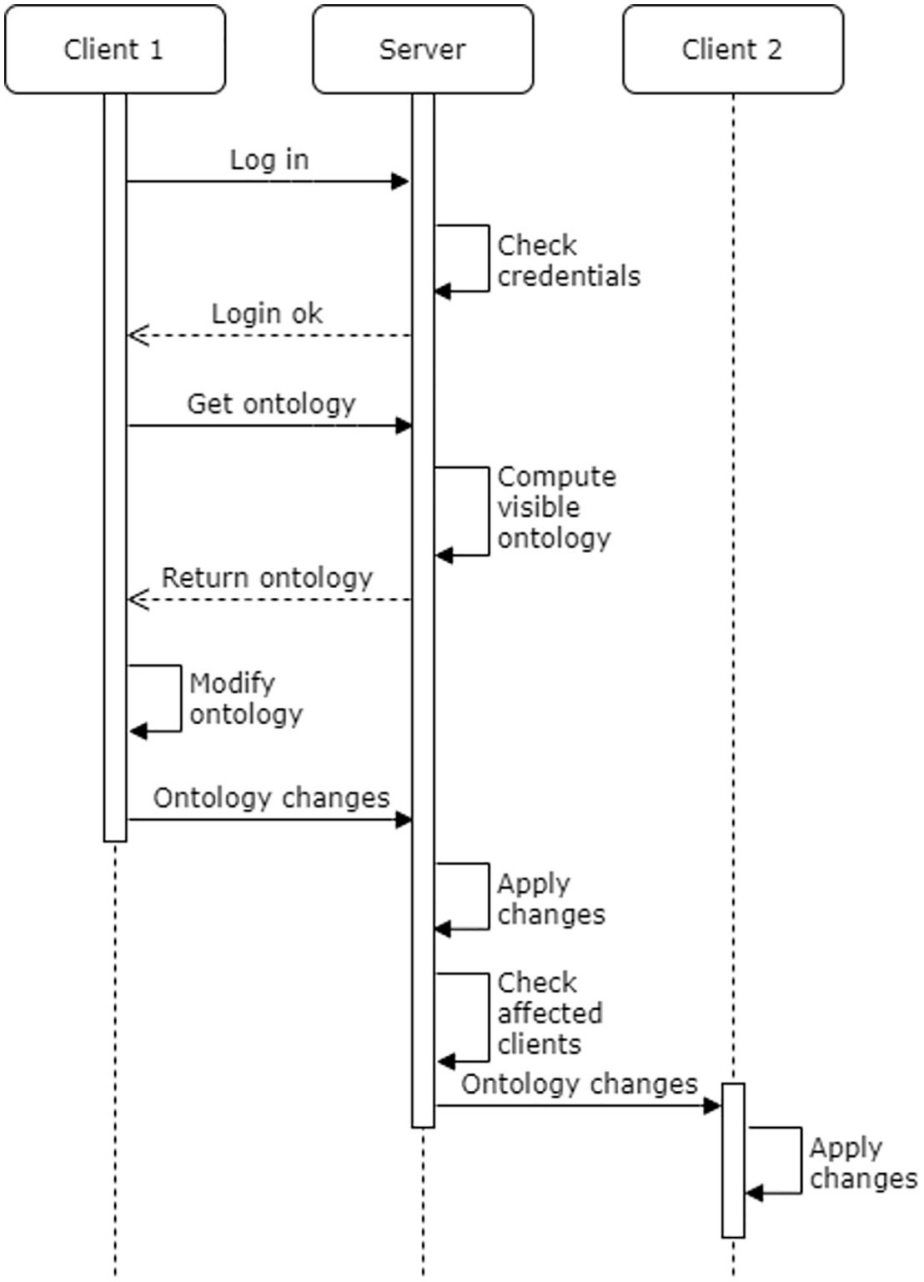
Ontology management. A sequence diagram depicting how the collaboration platform manages the underlying ontology. The server and the clients each hold a copy of the ontology; the server is responsible for ensuring that these remain synchronized as clients modify their local copies.

The client component consists of a software application and a local database, which is used to store the contents of datasets created or downloaded by the user. When a user logs in, the client initially receives from the server a copy of the master ontology, pruned to contain only those parts that are visible to the user (e.g. only those collaborations that the user is participating in or has been invited to). Ontology changes made by the client are first applied to the local copy, then sent to the server where they are applied to the master copy, and finally forwarded to other connected clients to be applied to their respective local copies. The ontology is thus managed in a distributed manner by the server and all active clients as illustrated in Fig 3.

When a user of the platform creates a new dataset, initially an empty one is created and recorded as an individual in the ontology with a name and a description attached to it as data properties. When the user populates the dataset with data, subsets and data items representing the structure and content of the dataset are created. These are also recorded as individuals in the ontology and linked to the dataset and to one another using the appropriate object properties. The individuals representing data items have the corresponding variable values attached to them as data properties.

All of the above information except the data item values is transmitted to the server and becomes visible to other users participating in collaborations where the dataset is used. The server makes the information persistent by storing the ontology on disk on the server host. The actual data contained by the dataset is stored in the client’s local database under a unique identifier generated by the software platform. When a user logs in to the server, information about the user’s datasets is extracted from the ontology, and the datasets are then populated with data retrieved from the local database, if available.

When a dataset is added to a collaboration, the corresponding ontology individuals are linked together by the appropriate object property. All users participating in the collaboration will then have access to metadata about the dataset and can request the owner of the dataset to grant them access to its contents. When a data request is granted by the data owner, applicable privacy constraints are first evaluated to identify any parts of the dataset to be excluded from being shared. The values of data items not excluded are then uploaded to the server, which will maintain a temporary copy of the data until the sender of the data request downloads it. Once the download is complete, the server will delete the temporary copy.

The creation of an analysis result by an expert triggers a variation of the basic data sharing process. The result dataset is linked to the original dataset by the appropriate object property, establishing a derivative-of relationship between them, and the creation of a data request is skipped, since the owner of the original is inferred by the reasoner to also control the derivative. When the derivative dataset is populated, the data is immediately uploaded, without any constraints, to the server, from where the owner of the original dataset can download it. Once the download is complete, the data will again be deleted from the server.

**Fig 4:**
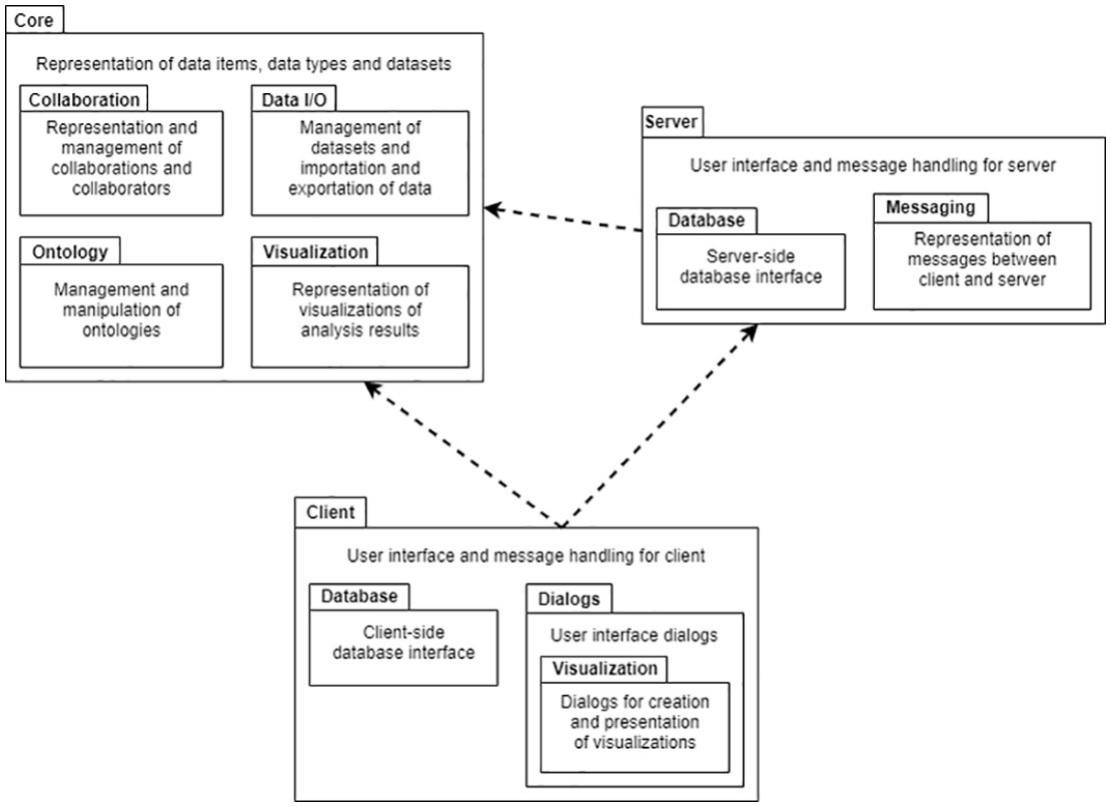
Software architecture. Modules and packages of the collaboration platform. Arrows indicate dependency relationships between modules.

The code base of the platform is divided into three main modules: Core, Server and Client. The Core module consists of packages on which both the other two modules depend, providing representations of essential collaboration concepts as well as ontology processing and data management functionality. Each main module contains a number of submodules dedicated to specific purposes; the dependency relationships and internal structure of the modules are illustrated in Fig 4.

The platform is implemented in the Java language. Apart from packages included in the standard Java API, the implementation depends on the following libraries:

- Apache MINA for client-server communications over TCP/IP;
- OWL API for ontology processing;
- Apache Commons CSV for data import/export;
- XChart for displaying visualizations;
- Nitrite for client-side database management;
- MiG Layout for graphical user interfaces.

The server-side database of the platform is implemented using the MongoDB database management system. Apart from MongoDB, the platform software is completely standalone, requiring no other software to be installed in order to be fully functional.

## Evaluation

### Fulfillment of requirements

Although the collaboration platform does not currently offer some of the more advanced functionality envisioned under Tasks and challenges, it does have all of the key features listed under Key requirements. Furthermore, through the combination of software logic and domain ontology, the platform fulfills all three special data management requirements identified earlier:

- **No centralized storage**: Each client manages its own datasets and stores them in a local Nitrite database. The server is only used for temporary storage of data in the process of being shared with a collaborator.
- **Case-by-case basis**: Privacy constraints can be specified separately for each time a dataset is requested to be shared; the effects of the constraints are displayed for the data owner to review before granting the request. The ontology provides classes for representing privacy-preserving data transformations and their effects on data utility.
- **Maintaining dataset provenance**: By following the chain of ontology object properties representing how datasets have been derived from other datasets, each dataset recorded in the ontology can be traced back to the original dataset created by the user who collected the data.

Not all of the features of the ontology are used by the current implementation of the software, more precisely the specification of analysis tasks and privacy constraints that prescribe a transformation of the data instead of simply blocking it, and data categories are also not yet implemented. Within these limitations, the collaboration process has been tested on volunteer participants in a real-world trial and shown to be feasible and potentially beneficial. Furthermore, the unused ontology features have been tested in the Protégé environment [67] using synthetic data to confirm that the following inferences are correctly made:

- DataItems are inferred to be members of the correct DataGroupings based on asserted subgrouping relationships;
- Conflicts between AnalysisTasks and PrivacyConstraints (i.e. DataItems that are both required by an AnalysisTask and restricted by a PrivacyConstraint) are detected;
- Output Datasets generated by AnalysisTasks are inferred to be derivatives of the correct input Datasets.

### User trial

#### Scenario and arrangements

For the user trial, 13 volunteer participants were recruited by distributing a call for participation via an institutional mailing list. The use of human subjects was approved by the Research Ethics Committee (REC) of Dublin City University, and written consent was obtained from each human subject participating in the study. Out of the initial 13, one participant elected to drop out of the study at an early stage. Each participant was offered a wearable health tracker on loan for the duration of the study; the models available were Fitbit Alta HR, Withings Steel and Ōura Ring. Participants who already had a suitable device were given the option of using their own.

The study participants were requested to wear their device for a period of approximately two months, preferably every night in order to capture as much of their sleep data as possible. They were also given instructions to export the data and use the collaboration platform to share it with an expert to have it analyzed.

Domain experts in the collaborations received data as it was shared by participants and studied it using periodicity analysis. Lomb-Scargle periodograms [68] were used to detect regularly repeating cycles in the data, such as the weekly periodicity commonly found in the sleeping habits of individuals who work a regular 5-day week. Furthermore, for those participants who provided enough data, periodicity intensity analysis was used to quantify the strength of such patterns and to track how it varied over the course of the observation period. This regularity of behaviour, including sleep, and its conformance with the 24 hour circadian rhythm has been shown in other work, to correlate with overall health and wellness [69].

To illustrate the analysis results, line graph visualizations were used to present the participants with graphical representations of their periodograms and periodicity intensity curves. Explanations of the visualizations were given using the chat function of the platform, and participants were encouraged to use the chat to ask any questions they might have. Having thus completed the collaboration, participants were requested to respond to a survey on their experience with the software platform and the collaboration process.

#### Execution and results

The trial was carried out between the beginning of October 2019 and the end of January 2020. During this time, participants were recruited in two stages. In an initial pilot stage, 3 volunteers began collecting data and using the platform to help reveal any software defects or other issues that needed to be addressed before proceeding any further with the trial. Approximately one month after the beginning of the pilot stage, the remaining volunteers were included in the trial.

To encourage the participants to share their sleep data and to view their analysis results, occasional email reminders were sent out over the course of the trial period. Out of the 12 volunteers who completed the trial, 8 did all the tasks they were requested to do, 10 shared at least one dataset with the researchers, and all 12 used the client application. All 12 also completed the survey. The survey questions are provided as supporting information in S1 File.

The survey was divided into four sections: background information, application usage, numeric evaluation and verbal feedback. In the background section, the respondents were requested to provide their gender and age and to evaluate their previous experience in lifelogging (defined as “using technology to capture data about yourself”) and knowledge discovery (defined as “using computational analysis techniques to extract knowledge from data”). Answers to the self-evaluation questions were given on a discrete scale of 1 to 5, where 1 signified “little or no previous experience” and 5 signified “highly experienced”. The responses to the background section are shown in Tables 2 and 3.

**Table 2:** Number of survey respondents by gender and age.

Gender	Respondents	Age	Respondents
Female	3	18–25	3
Male	9	26–35	6
		36–45	3

**Table 3:** Number of survey respondents by level of previous experience in lifelogging and knowledge discovery.

Subject	1	2	3	4	5	Avg
Lifelogging	4	1	2	4	1	2.75
Knowledge discovery	1	4	2	3	2	3.08

The respondents were asked to self-rate their level of experience on a discrete 1–5 scale where 1 = little or no experience, 5 = highly experienced.

In the application usage section of the survey, the respondents were first requested to answer a yes/no question on whether they used the application, which was defined as having logged in successfully at least once. All 12 respondents answered yes. They were then requested to answer 14 follow-up questions on whether they used various features of the application; the response options for these questions were “yes”, “no” and “I’m not sure”. The responses are shown in Table 4.

**Table 4:** Number of survey respondents by whether or not they used specific features of the application.

Feature	Yes	No	Not sure
Created a collaboration	11	1	0
Created a dataset	11	1	0
Imported data into a dataset	10	2	0
Added a dataset to a collaboration	10	2	0
Searched for experts	9	3	0
Sent an invitation to an expert	9	3	0
Added an expert to a collaboration	10	2	0
Reviewed a data request	9	3	0
Granted a data request	9	3	0
Attached privacy constraints to a data request	3	8	1
Downloaded analysis results	9	3	0
Viewed a visualization	9	3	0
Opened the chat window	9	3	0
Sent a chat message	6	4	2

In the numeric evaluation section of the survey, the respondents were given 10 statements about the application and the collaboration process. For each of these statements, they were requested to rate their agreement on a discrete scale of 1 to 5, where 1 signified “strongly disagree” and 5 signified “strongly agree”. Additionally, “N/A” was provided as a response that the respondents could select for statements that did not apply to them (for example, if the respondent had not used a particular feature of the application). The responses are shown in Table 5.

**Table 5:** Number of survey respondents by level of agreement with various statements.

Statement	1	2	3	4	5	N/A	Avg
The application was suitable for its purpose.	1	0	3	6	2	0	3.67
The application worked reliably.	0	5	4	3	0	0	2.83
The application was easy to use.	1	6	3	2	0	0	2.50
I would have preferred the application to run in a Web browser.	1	0	4	2	5	0	3.83
The collaboration process was easy to understand.	0	3	3	5	1	0	3.33
I found the analysis results interesting.	0	0	3	3	5	1	4.18
I learned something useful from the analysis results.	1	1	2	4	3	1	3.64
I would be willing to engage in this type of collaboration in the future.	2	0	1	5	3	1	3.64
I would be willing to share my data with an expert I don’t know well.	1	1	1	2	5	2	3.90
I would be willing to pay money to have someone help me analyse my data.	3	2	4	2	0	1	2.45

The respondents were asked to rate their level of agreement on a discrete 1–5 scale where 1 = strongly disagree, 5 = strongly agree. For statements that did not apply to them, the respondents were instructed to choose the N/A option.

Finally, in the verbal feedback section of the survey, the respondents were requested to answer the following open-ended questions:

1. Are there any general impressions concerning the application and/or the collaboration process that you would like to share?
2. Are there any problems with the application and/or the collaboration process that you would like to point out?
3. Are there any improvements to the application and/or the collaboration process that you would like to suggest?

The complete set of answers to these questions is provided as supporting information in S2 File. In the answers, the following themes were brought up in multiple responses:

- Broad themes:
  - The collaboration workflow and/or the user interface of the software should be more user-friendly.
  - The software is sometimes unresponsive and/or unstable (freezes / loses connection / fails to transfer data).
- Concrete suggestions:
  - The software should have a notification system to let the user know about new events requiring their attention.
  - Users should be provided with better instructions, illustrated with screenshots or a screencap video.

The implications of these results are discussed in the following section.

## Discussion

The successful completion of the trial shows that the ontology-based solution is feasible, at least on a small scale. One issue that does require further work is representation of the low-level structure of datasets: encoding the structure in the ontology all the way down to the level of individual data items was found to be impracticable in a real-world scenario, and even after this problem was circumvented, datasets with a large number of variables were found to be problematic. A more efficient protocol for transmitting this information would help considerably in eliminating the problem.

The main purpose of the trial was to collect data on how users, specifically data owners, experience the software and the collaboration process and how it might be improved. Self-reporting of application usage (Table 4) was requested in order to obtain additional background information for these experiences, with particular focus on revealing aspects of the software that the users may have been confused about. It is worth noting that those who shared data also downloaded analysis results and viewed visualizations, meaning that in the numeric evaluation and verbal feedback questions, the answers of most respondents are based on experiences of at least one full execution of the collaboration cycle.

The least used features of the application were creating a privacy constraint and sending a chat message, for which a likely explanation is that completing the collaboration cycle did not require these. The option to create constraints was available when responding to a data request, but most users were content with the default option of sharing their dataset in its entirety. Users were encouraged to use the chat feature to engage in a dialogue with the expert collaborator on the significance of the analysis results, but many were content with reading the expert’s explanations from the chat without responding to them.

The collaboration concept was well received, with a particularly strong agreement on the statement that the analysis results were interesting. The majority also agreed that they learned something useful and that they would be willing to engage in similar collaborations in the future. In addition to expressing an interest in engaging in future collaborations, the respondents indicated that they would be willing to share their data with an expert they do not know well, further corroborating the viability of the collaboration concept. However, their willingness to pay the expert for helping them analyze the data was fairly low on average. If the expert is not receiving a monetary compensation, some other kind of incentive is likely to be required; depending on the interests of the expert, in some cases the data and the analysis itself may be a sufficient incentive.

Considering the small size of the group of users recruited for the trial, a good mix of different genders and age groups was achieved. Moreover, the users did not, on average, have a particularly high level of relevant technical expertise. However, further research is necessary in order to find out to what degree the above results are generalizable.

Concerning the data management requirements, the platform does not currently provide end-to-end encryption for data being shared with a collaborator; this is not one of the key requirements, but clearly it would be preferable from a privacy standpoint. Another desirable feature would be synchronization of the local database across client instances to enable a user to conveniently access the collaboration platform from multiple devices. Both these issues are among the top short-term priorities in future development of the platform.

In the medium to long term, future work will focus on expanding the ontology and developing new software functionality to make full use of the knowledge representation and reasoning capabilities provided by the ontology. A particularly important research challenge is representing detailed and actionable knowledge about data transformation methods, both those used for data analysis and those used for privacy preservation, and about the effects that the latter have on the utility of the former. Another important research direction would be to analyze related ontologies in order to identify those that could be imported into the collaboration ontology to expand its scope.

## Conclusion

In this paper we introduced a new software platform designed to enable individuals with no data analysis expertise to extract knowledge from their personal data by collaborating with expert data analysts. Unlike existing collaborative data analysis systems, the platform emphasizes negotiation of collaboration terms and collaborative evaluation of analysis results over data analysis per se, since these are more important from the non-expert data owner’s perspective. Users of the platform can search for potential collaborators and invite them to collaborate, communicate with one another by text-based chat, exchange input datasets and analysis results, and create and view visualizations of results to support interpretation and evaluation.

The platform is implemented as a Java-based client-server system with an underlying domain ontology providing essential knowledge representation and reasoning capabilities. To validate the platform, a trial was carried out, with 12 volunteers collecting sleep data using wearable devices and analyzing it in collaboration with an expert. The results of the trial demonstrate the viability of the collaborative personal analytics concept and the feasibility of the ontology-based architecture of the platform.

## Supporting information

S1 File User test feedback survey. The participants of the user test were requested to respond to this survey after completion of the test.
(PDF)

S2 File Answers to open-ended survey questions. The complete set of answers submitted by the user test participants to the three open-ended questions included in the feedback survey.
(PDF)

## References

[1] Feng S, Mäntymäki M, Dhir A, Salmela H. How Self-tracking and the Quantified Self Promote Health and Well-being: Systematic Review. Journal of Medical Internet Research. 2021;23(9):e25171. doi: 10.2196/25171 34546176 PMC8493454

[2] Rooksby J, Asadzadeh P, Rost M, Morrison A, Chalmers M. Personal Tracking of Screen Time on Digital Devices. In: Proceedings of the 2016 CHI Conference on Human Factors in Computing Systems; 2016. p. 284–296.

[3] Spiekermann S, Acquisti A, Böhme R, Hui KL. The challenges of personal data markets and privacy. Electronic Markets. 2015;25(2):161–167.

[4] Malgieri G, Custers B. Pricing privacy—the right to know the value of your personal data. Computer Law & Security Review. 2018;34(2):289–303. doi: 10.1016/j.clsr.2017.08.006

[5] Tikkinen-Piri C, Rohunen A, Markkula J. EU General Data Protection Regulation: Changes and implications for personal data collecting companies. Computer Law & Security Review. 2018;34(1):134–153. doi: 10.1016/j.clsr.2017.05.015

[6] Gurrin C, Smeaton AF, Doherty AR. LifeLogging: Personal Big Data. Foundations and Trends in Information Retrieval. 2014;8(1):1–125. doi: 10.1561/1500000033

[7] Swan M. The Quantified Self: Fundamental Disruption in Big Data Science and Biological Discovery. Big Data. 2013;1(2):85–99. doi: 10.1089/big.2012.0002 27442063

[8] Microsoft Research. SenseCam; 2004. Available from: https://www.microsoft.com/en-us/research/project/sensecam/.

[9] Vogels EA. About one-in-five Americans use a smart watch or fitness tracker; 2020. Available from: https://www.pewresearch.org/fact-tank/2020/01/09/about-one-in-five-americans-use-a-smart-watch-or-fitness-tracker/.

[10] Al-Naser A, Rasheed M, Irving D, Brooke J. A Visualization Architecture for Collaborative Analytical and Data Provenance Activities. In: 2013 17th International Conference on Information Visualisation; 2013. p. 253–262.

[11] Hedayati N, Khademi M. A proposed architecture for collaborative data visualization systems. In: 2014 4th International Conference on Computer and Knowledge Engineering (ICCKE); 2014. p. 123–127.

[12] Li J, Chou JK, Ma KL. High performance heterogeneous computing for collaborative visual analysis. In: SIGGRAPH Asia 2015 Visualization in High Performance Computing; 2015. p. 12:1–12:4.

[13] Nguyen H, Marendy P, Engelke U. Collaborative framework design for immersive analytics. In: 2016 Big Data Visual Analytics (BDVA); 2016.

[14] Langner R, Horak T, Dachselt R. VisTiles: Coordinating and combining co-located mobile devices for visual data exploration. IEEE Transactions on Visualization and Computer Graphics. 2018;24(1):626–636. doi: 10.1109/TVCG.2017.2744019 28866515

[15] Chung H, North C, Self JZ, Chu S, Quek F. VisPorter: Facilitating information sharing for collaborative sensemaking on multiple displays. Personal and Ubiquitous Computing. 2014;18(5):1169–1186. doi: 10.1007/s00779-013-0727-2

[16] Donalek C, Djorgovski SG, Cioc A, Wang A, Zhang J, Lawler E, et al. Immersive and collaborative data visualization using virtual reality platforms. In: 2014 IEEE International Conference on Big Data (Big Data); 2014. p. 609–614.

[17] Moraes AC, Eler DM, Brega JRF. Collaborative information visualization using a multi-projection system and mobile devices. In: 2014 18th International Conference on Information Visualisation; 2014. p. 71–77.

[18] Bhojwani S, Hemmings M, Ingalls D, Lincke J, Krahn R, Lary D, et al. The Ignite distributed collaborative scientific visualization system. In: 2015 IEEE 7th International Conference on Cloud Computing Technology and Science (CloudCom); 2015. p. 186–191.

[19] Jeong DH, Ji SY, Suma EA, Yu B, Chang R. Designing a collaborative visual analytics system to support users’ continuous analytical processes. Human-centric Computing and Information Sciences. 2015;5(5).

[20] Su S, Perry V, Cantner N, Kobayashi D, Leigh J. High-resolution interactive and collaborative data visualization framework for large-scale data analysis. In: 2016 International Conference on Collaboration Technologies and Systems (CTS); 2016. p. 275–280.

[21] Gonzalez H, Halevy AY, Jensen CS, Langen A, Madhavan J, Shapley R, et al. Google Fusion Tables: Web-centered data management and collaboration. In: Proceedings of the 2010 ACM SIGMOD International Conference on Management of Data; 2010. p. 1061–1066.

[22] Howe B, Halperin D, Ribalet F, Chitnis S, Armbrust EV. Collaborative science workflows in SQL. Computing in Science & Engineering. 2013;15(3):22–31. doi: 10.1109/MCSE.2013.42

[23] Bhardwaj A, Karger D, Subramanyam H, Deshpande A, Madden S, Wu E, et al. Collaborative data analytics with DataHub. Proceedings VLDB Endowment. 2015;8(12):1916–1919. doi: 10.14778/2824032.2824100 26844007 PMC4734646

[24] Chourasia A, Wong M, Mishin D, Nadeau DR, Norman M. SeedMe: A scientific data sharing and collaboration platform. In: Proceedings of the XSEDE16 Conference on Diversity, Big Data, and Science at Scale; 2016. p. 48:1–48:6.

[25] North S, Scheidegger C, Urbanek S, Woodhull G. Collaborative visual analysis with RCloud. In: 2015 IEEE Conference on Visual Analytics Science and Technology (VAST); 2015. p. 25–32.

[26] Diamantini C, Potena D, Storti E. A virtual mart for knowledge discovery in databases. Information Systems Frontiers. 2013;15(3):447–463. doi: 10.1007/s10796-012-9399-0

[27] Diamantini C, Potena D, Storti E. Collaborative management of a repository of KDD processes. International Journal of Metadata, Semantics and Ontologies. 2014;9(4):299–311. doi: 10.1504/IJMSO.2014.065428

[28] Park K, Nguyen MC, Won H. Web-based collaborative big data analytics on big data as a service platform. In: 2015 17th International Conference on Advanced Communication Technology (ICACT); 2015. p. 564–567.

[29] Chin G, Lansing CS. Capturing and supporting contexts for scientific data sharing via the biological sciences collaboratory. In: Proceedings of the 2004 ACM Conference on Computer Supported Cooperative Work; 2004. p. 409–418.

[30] Karacapilidis N, Christodoulou S, Tzagarakis M, Tsiliki G, Pappis C. Strengthening collaborative data analysis and decision making in web communities. In: Proceedings of the 23rd International Conference on World Wide Web; 2014. p. 1005–1010.

[31] Jalili V, Afgan E, Gu Q, Clements D, Blankenberg D, Goecks J, et al. The Galaxy platform for accessible, reproducible and collaborative biomedical analyses: 2020 update. Nucleic Acids Research. 2020;48(W1):W395–W402. doi: 10.1093/nar/gkaa434 32479607 PMC7319590

[32] Kiddle C, Taylor AR, Cordes J, Eymere O, Kaspi V, Pigat D, et al. CyberSKA: an on-line collaborative portal for data-intensive radio astronomy. In: Proceedings of the 2011 ACM workshop on Gateway computing environments; 2011. p. 65–72.

[33] Erdmann M, Fischer R, Glaser C, Klingebiel D, Komm M, Müller G, et al. A web-based development environment for collaborative data analysis. Journal of Physics: Conference Series. 2014;523(1):012021.

[34] Chen X, Madhavan K, Vorvoreanu M. A web-based tool for collaborative social media data analysis. In: 2013 International Conference on Cloud and Green Computing; 2013. p. 383–388.

[35] Marée R, Rollus L, Stévens B, Hoyoux R, Louppe G, Vandaele R, et al. Collaborative analysis of multi-gigapixel imaging data using Cytomine. Bioinformatics. 2016;32(9):1395–1401. doi: 10.1093/bioinformatics/btw013 26755625 PMC4848407

[36] Chine K. Scientific computing environments in the age of virtualization: Toward a universal platform for the cloud. In: 2009 IEEE International Workshop on Open-source Software for Scientific Computation (OSSC); 2009. p. 44–48.

[37] Mode Analytics, Inc. Mode website; 2020. Available from: https://mode.com.

[38] Baglietto P, Fornasa M, Maresca M, Stecca M. Collaboration and real-time analysis in the SpreadSheet Space. In: Proceedings of the 17th International Conference on Information Integration and Web-based Applications & Services; 2015. p. 37.

[39] Kandogan E, Roth M, Schwarz P, Hui J, Terrizzano I, Christodoulakis C, et al. LabBook: Metadata-driven social collaborative data analysis. In: 2015 IEEE International Conference on Big Data (Big Data); 2015. p. 431–440.

[40] Apache Software Foundation. Apache Zeppelin website; 2020. Available from: http://zeppelin.apache.org.

[41] Dataiku. Dataiku website; 2020. Available from: https://www.dataiku.com.

[42] Databricks. Collaborative Notebooks; 2020. Available from: https://databricks.com/product/collaborative-notebooks.

[43] Fumarola F, Salvemini E, Malerba D. A KDD platform based on the application service provider paradigm. In: IEEE International Conference on Data Mining Workshops, 2008. ICDMW’08.; 2008. p. 983–986.

[44] Diamantini C, Potena D, Storti E. A semantic-aided designer for knowledge discovery. In: 2011 International Conference on Collaboration Technologies and Systems (CTS); 2011. p. 86–93.

[45] Deshpande M, Ray D, Dixit S, Agasti A. ShareInsights: An unified approach to full-stack data processing. In: Proceedings of the 2015 ACM SIGMOD International Conference on Management of Data; 2015. p. 1925–1940.

[46] Sage Bionetworks. Synapse website; 2020. Available from: https://www.synapse.org.

[47] Korpela EJ. SETI@home, BOINC, and volunteer distributed computing. Annual Review of Earth and Planetary Sciences. 2012;40:69–87. doi: 10.1146/annurev-earth-040809-152348

[48] Gaber MM, Stahl F, Gomes JaB. Pocket Data Mining: Big Data on Small Devices. vol. 2 of Studies in Big Data. Cham: Springer; 2014.

[49] Clery D. Galaxy Zoo volunteers share pain and glory of research. Science. 2011;333(6039):173–175. doi: 10.1126/science.333.6039.173 21737731

[50] Mavandadi S, Dimitrov S, Feng S, Yu F, Sikora U, Yaglidere O, et al. Distributed medical image analysis and diagnosis through crowd-sourced games: A malaria case study. PLOS ONE. 2012;7(5):1–8. doi: 10.1371/journal.pone.0037245 22606353 PMC3350488

[51] Tuovinen L, Smeaton AF. Remote Collaborative Knowledge Discovery for Better Understanding of Self-tracking Data. In: Proceedings of the 25th Conference of Open Innovations Association FRUCT; 2019. p. 324–332.

[52] Panov P, Soldatova L, Džeroski S. OntoDM-KDD: Ontology for Representing the Knowledge Discovery Process. In: Proceedings of the 16th International Conference on Discovery Science; 2013. p. 126–140.

[53] Panov P, Soldatova L, Džeroski S. Ontology of Core Data Mining Entities. Data Mining and Knowledge Discovery. 2014;28(5-6):1222–1265. doi: 10.1007/s10618-014-0363-0

[54] Panov P, Soldatova LN, Džeroski S. Generic Ontology of Datatypes. Information Sciences. 2016;329:900–920. doi: 10.1016/j.ins.2015.08.006

[55] Kietz JU, Serban F, Fischer S, Bernstein A. “Semantics Inside!” But Let’s Not Tell the Data Miners: Intelligent Support for Data Mining. In: The Semantic Web: Trends and Challenges, 11th International Conference, ESWC 2014, Proceedings; 2014. p. 706–720.

[56] Keet CM, Ławrynowicz A, d’Amato C, Kalousis A, Nguyen P, Palma R, et al. The Data Mining OPtimization Ontology. Web Semantics: Science, Services and Agents on the World Wide Web. 2015;32:43–53. doi: 10.1016/j.websem.2015.01.001

[57] Kumara BTGS, Paik I, Zhang J, Siriweera THAS, Koswatte KRC. Ontology-Based Workflow Generation for Intelligent Big Data Analytics. In: Proceedings of the 2015 IEEE International Conference on Web Services; 2015. p. 495–502.

[58] Li Y, Thomas MA, Osei-Bryson KM. Ontology-based Data Mining Model Management for Self-service Knowledge Discovery. Information Systems Frontiers. 2017;19(4):925–943. doi: 10.1007/s10796-016-9637-y

[59] Allison DS, Kamoun A, Capretz MAM, Tazi S, Drira K, ElYamany HF. An Ontology Driven Privacy Framework for Collaborative Working Environments. International Journal of Autonomous and Adaptive Communications Systems. 2016;9(3-4):243–268. doi: 10.1504/IJAACS.2016.079624

[60] Bhatia R, Singh M. Privacy Issues in Web Services: An Ontology Based Solution. Procedia Computer Science. 2016;92:461–467. doi: 10.1016/j.procs.2016.07.368

[61] Banerjee A, Joshi KP. Link Before You Share: Managing Privacy Policies through Blockchain. In: Proceedings of the 2017 IEEE International Conference on Big Data; 2017. p. 4438–4447.

[62] Gharib M, Giorgini P, Mylopoulos J. Towards an Ontology for Privacy Requirements via a Systematic Literature Review. In: Conceptual Modeling, 36th International Conference, ER 2017, Proceedings; 2017. p. 193–208.

[63] Ghorbel A, Ghorbel M, Jmaiel M. PRIARMOR: An IaaS Solution for Low-level Privacy Enforcement in the Cloud. In: Proceedings of the 2017 IEEE 26th International Conference on Enabling Technologies: Infrastructure for Collaborative Enterprises; 2017. p. 119–124.

[64] Oltramari A, Piraviperumal D, Schaub F, Wilson S, Cherivirala S, Norton TB, et al. PrivOnto: A Semantic Framework for the Analysis of Privacy Policies. Semantic Web. 2018;9(2):185–203. doi: 10.3233/SW-170283

[65] Sanchez OR, Torre I, Knijnenburg BP. Semantic-based Privacy Settings Negotiation and Management. Future Generation Computer Systems. 2019.

[66] Tuovinen L, Smeaton AF. A Domain Ontology and Software Platform for Collaborative Personal Data Analytics. In: Proceedings of the 16th International Conference on Cooperative Design, Visualization, and Engineering; 2019. p. 1–10.

[67] Musen MA, Protégé Team. The Protégé Project: A Look Back and a Look Forward. AI Matters. 2015;1(4):4–12. doi: 10.1145/2757001.2757003 27239556 PMC4883684

[68] Ruf T. The Lomb-Scargle periodogram in biological rhythm research: analysis of incomplete and unequally spaced time-series. Biological Rhythm Research. 1999;30(2):178–201. doi: 10.1076/brhm.30.2.178.1422 11708361

[69] Buman MP, Hu F, Newman E, Smeaton AF, Epstein DR. Behavioral Periodicity Detection from 24 h Wrist Accelerometry and Associations with Cardiometabolic Risk and Health-Related Quality of Life. BioMed research international. 2016;2016(Article ID 4856506):9. doi: 10.1155/2016/4856506 26942195 PMC4752978

