# Supplementary material for: Privacy-aware sharing and collaborative analysis of personal wellness data: Process model, domain ontology, software system and user trial
Source: PLoS One. 2022 Apr 7;17(4):e0265997. doi: 10.1371/journal.pone.0265997 (PMC8989328; doi:10.1371/journal.pone.0265997)
Supplement: S1 File — The participants of the user test were requested to respond to this survey after completion of the test. (PDF) [file pone.0265997.s001.pdf]

Supporting information: User test feedback survey

Background

Gender
Options: Female | Male | Prefer not to say

Age
Options: 18-25 | 26-35 | 36-45 | 46-55 | 56-65 | 66 or older

Before participating in the study, how much experience did you have in the following subjects? 1 = little or no previous experience, 5 = highly experienced.

- Lifelogging (using technology to capture data about yourself)
- Knowledge discovery (using computational analysis techniques to extract knowledge from data)

Options: 1 | 2 | 3 | 4 | 5

Application usage

The questions in this section record your usage of the collaborative data analysis software application.

Did you use the application? Answer yes if you logged in successfully at least once.
Options: Yes | No

If you used the application, did you do any of the following? If you didn't use the application, select no for each row.

- Created a collaboration
- Created a dataset
- Imported data into a dataset
- Added a dataset to a collaboration
- Searched for experts
- Sent an invitation to an expert
- Added an expert to a collaboration

Options: Yes | No | I'm not sure

If you created a collaboration, did you do any of the following? If you didn't create a collaboration, select no for each row.

- Reviewed a data request
- Granted a data request
- Attached privacy constraints to a data request
- Downloaded analysis results
- Viewed a visualisation
- Opened the chat window
- Sent a chat message

Options: Yes | No | I'm not sure

Feedback

The questions in this section record your opinions on the software application and the collaboration process.

Do you agree with the following statements? 1 = strongly disagree, 5 = strongly agree. If a statement doesn't apply in your case, select N/A.

- The application was suitable for its purpose.
- The application worked reliably.
- The application was easy to use.
- I would have preferred the application to run in a Web browser.
- The collaboration process was easy to understand.
- I found the analysis results interesting.
- I learned something useful from the analysis results.
- I would be willing to engage in this type of collaboration in the future.
- I would be willing to share my data with an expert I don't know well.
- I would be willing to pay money to have someone help me analyse my data.

Options: 1 | 2 | 3 | 4 | 5 | N/A

Are there any general impressions concerning the application and/or the collaboration process that you would like to share?

Are there any problems with the application and/or the collaboration process that you would like to point out?

Are there any improvements to the application and/or the collaboration process that you would like to suggest?
