# Supplementary material for: Privacy-aware sharing and collaborative analysis of personal wellness data: Process model, domain ontology, software system and user trial
Source: PLoS One. 2022 Apr 7;17(4):e0265997. doi: 10.1371/journal.pone.0265997 (PMC8989328; doi:10.1371/journal.pone.0265997)
Supplement: S2 File — The complete set of answers submitted by the user test participants to the three open-ended questions included in the feedback survey. (PDF) [file pone.0265997.s002.pdf]

Supporting information: Answers to open-ended survey questions

Places where a reference to an individual person has been anonymised are indicated by square brackets. Apart from anonymisation, the answers are presented exactly as submitted by the user test participants.

Are there any general impressions concerning the application and/or the collaboration process that you would like to share?

- I found the pipeline for the collaboration a bit confusion, I think it can be more user friendly.
- User interface could be simpler
- This application is not complicated if knowing how to use in advance. But the process and interface are not friendly.
- I think the idea behind this is very interesting as sleep seems to impact on every part of your life- maybe some research around this could be highlighted at some point in the feedback process.
- I like the idea of sharing my sleep data with experts that give me some insights. The workflow made sense, from creating datasets, to sharing them and getting outputs
- Portal was difficult to use
- not really
- Had difficulty opening and running the application

Are there any problems with the application and/or the collaboration process that you would like to point out?

- I didn't like to wait for the expert to accept. I would like to open the application only one time per dataset and then receive the results via email, for example.
- In my case if I leave the app open at some point it lose connection and it freeze.
- Some moments the application was a bit confusing.
- Occasionally lagginess and unresponsiveness
- The application is not stable and always arises losing connection.
- A couple of times the upload crashed. Perhaps have a look at how used friendly it is- it is not as intuitive as it could be to use the app, or if you are unable to change the app perhaps more clearly lay out the instructions in clear, short bullet points with screenshots of the process, or create a screengrab video participants can follow along with. A trouble shooting guide would be useful in response to common bugs.
- Application failed to connect or download data from the server sometimes but [researcher] was always there to correct it or find a workaround
- Portal was difficult to use
- have to read the instruction all the time
- There were issues with getting the application to work. I successfully logged in 3 times but every time I tried to load my data it told me to come back later that it couldn't load at that time. And then I wouldn't have a chance to come back for some time.

Are there any improvements to the application and/or the collaboration process that you would like to suggest?

- I would design a more user friendly interface and explain better what is the visualization displaying, like in the Oura app.
- Join the process of creating the collaboration and uploading the first dataset in one step (one form), it would make more sense for the user
- Some notification system that tells you when the process is stopped because of you (when you have to grant access to the data or other) or when the expert send you a message...
- Hire a designer :)
- I suggest to provide guideline with screenshots or a use case for users, which would be easiler for them to use this application.
- Notifications would be useful to alert participants of a new message or time to log data. Perhaps a little more clarity would be useful with regard to 'periodicy' and how this may be relevant for day to day lives (e.g.practical implications- does a lack of consistent sleep cycle result in X, Y Z...) Maybe presenting norms and plotting each persons data against that would be useful (though I realise you probably don;t have norms quite yet).
- A web version would make sense
- Portal was difficult to use
- maybe instead of multiple windows combine into one GUI interface?
- More aesthetic UI
- The interaction needs to be made easier and more straightforward. If it was in an app or browser, when the specialist interacted or wanted answers to questions, a notification could come up. Needing to remember to log in every few days is not ideal. It would be better if there were notifications.
